# *Saccharina japonica* Ethanol Extract Ameliorates Depression/Anxiety-Like Behavior by Inhibiting Inflammation, Oxidative Stress, and Apoptosis in Dextran Sodium Sulfate Induced Ulcerative Colitis Mice

**DOI:** 10.3389/fnut.2021.784532

**Published:** 2021-12-16

**Authors:** Xiufang Dong, Kuan Lu, Pengcheng Lin, Hongxia Che, Hongyan Li, Lin Song, Xihong Yang, Wancui Xie

**Affiliations:** Shandong Provincial Key Laboratory of Biochemical Engineering, College of Marine Science and Biological Engineering, Qingdao University of Science and Technology, Qingdao, China

**Keywords:** *Saccharina japonica*, ulcerative colitis, depression, anxiety, brain

## Abstract

*Saccharina japonica* is a common marine vegetable in East Asian markets and has a variety of health benefits. This study was focused on the anti-depressant/anxiety effects of Saccharina japonica ethanol extract (SJE) on dextran sodium sulfate (DSS)-induced mice and its potential mechanism in their brain. Male C57BL/6 mice were treated with mesalazine and various doses of SJE (1, 2, and 4 g/kg body weight) for 2 weeks, followed by DSS treatment at the second week. The DSS-induced mice showed depression/anxiety-like behavior, which included shorter path length in the open field test and longer immobility time in the tail suspension test. L-SJE alleviated the depression-like behaviors. In the DSS-induced mice, reduced synaptic plasticity activated microglia, increased proinflammatory cytokines, decreased anti-inflammatory cytokine, and increased expression levels of Toll-like receptors-4, nuclear factor kappa-B, NOD-like receptors 3, apoptosis-associated speck-like protein, and Caspase-1 were observed, most of which were alleviated by SJE treatment. Furthermore, all the SJE groups could significantly enhance superoxide dismutase activity, while the L-SJE treatment decreased the contents of malondialdehyde, and the H-SJE treatment inhibited apoptosis. All these results showed that the SJE might serve as a nutritional agent for protecting the brain in ulcerative colitis mice.

## Introduction

Inflammatory bowel disease (IBD) is a chronic inflammatory disease, which is associated with a mental dysfunction, and severely affects the quality of patients' lives (1). Over 30% of the IBD patients have psychiatric disorders, including depression and anxiety (2). The might be due to the disruption of the microbe-gut-brain axis, caused by the bidirectional signaling of inflammatory mediators, metabolic signals, oxidative stress markers and modulators, and neurohormonal factors (3). Some plant extracts have been reported to alleviate chronic neurological diseases, induced by IBD, based on these signaling pathways (4).

Depression and anxiety are common neurological disorders, triggered by genetic, environmental, or stress factors. Their pathological mechanisms include the disturbance of neuroanalytic system, activation of immune/inflammatory responses, damage to neuroplasticity, disturbance of neurotransmitters, structural changes in brain, and disturbance of neural circuit (5). Depression/anxiety-like behaviors are accompanied by cerebral inflammation, cellular damage, and apoptosis. It has been widely reported that the up-regulated pro-inflammatory cytokines, such as tumor necrosis factor-α (TNF-α), interleukin-6 (IL-6) and IL-1β are related to depression/anxiety-like behaviors (6). The upregulation of these cytokines can be activated by inflammatory signaling pathways, such as toll-like receptors-4 (TLR4) and NOD-like receptors 3 (NLRP3) signaling pathways, which are the most relevant pathways to neurological disorders induced by external or internal stress factors (7). TLR4 can activate nuclear factor kappa-B (NF-κB) through a cascade of reactions and induce the NLRP3 inflammasome pathway, leading to the maturation of IL-1β (8). Some terrestrial plant extracts rich in phenol, flavonoids, and pyrazine compounds (9–11) and probiotics, such as *Lactobacillus delbrueckii* (12) can alleviate depression/anxiety-like behavior in the IBD mice by inhibiting the TLR4-NLRP3 signaling pathways. Nevertheless, the mechanism of marine algae extracts rich in phlorotannins and fucoxanthin on depression/anxiety-like behavior in the IBD mice was not reported.

During the coronavirus disease-19 (COVID-19) pandemic, there is also a growing concern about the impact of food on health. Several studies conducted on a variety of foods, including some fruit and vegetable raw materials (13), have stimulated the search for natural products. *Saccharina japonica*, also known as *Laminaria japonica* and is a common marine vegetable in East Asian markets, is the most highly-valued algae in China. *Saccharina japonica* ethanol extract (SJE) mainly contain active ingredients, such as phlorotannins, fucoxanthin and fucosterol, which exhibit favorable biological activities, such as antioxidant (14), anti-inflammatory (15), and anticancer activities (16). Furthermore, phenolic compounds and fucoxanthin could cross the blood-brain barrier and exert anti-psychiatric effects by acting on multiple targets, including neuroinflammation, oxidative stress, neurotransmission dysregulation, and disturbance of gut microbiota (17–19). SJ extracts have been shown to have biological effects such as anti-oxidation, improving memory, anti-cancer, inhibiting inflammation, and regulating lipid metabolism. However, little is known about the effects of SJ on DSS induced colitis and its complications.

Our previous study demonstrated that *Saccharina japonica* ethanol extract (SJE) exhibited a protective effect against dextran sulfate sodium (DSS)-induced ulcerative colitis (UC) by improving the integrity of intestinal barrier, inhibiting the apoptosis of colonic epithelial cells, regulating the production of colonic inflammatory cytokines and oxidative stress, and reshaping the gut microbiota Che et al. under review. However, the effects of SJE on depression/anxiety-like behavior in UC mice and the role of these effects in the inhibition of inflammation, oxidative stress, and apoptosis, remain unknown and require evaluation to determine these effects on UC-associated depression and anxiety. Therefore, this study aimed to study the protective effects of SJE against UC-associated depression/anxiety. First, the effects of SJE on behavioral disorders and synaptic plasticity in DSS-induced mice were evaluated. Subsequently, the effects of SJE on alleviating depression and anxiety by inflammatory response were investigated by measuring the expression levels of inflammatory cytokines and key proteins in TLR4/NLRP3 signaling pathways. Finally, the effects of SJE on inhibiting the oxidative stress and apoptosis in DSS-induced mice were evaluated.

## Materials and Methods

### Materials and Chemicals

*Saccharina japonica* was purchased from Kaiping Road Market in Qingdao. DSS (molecular weight 36–50 kDa) was purchased from MP Biomedicals LLC. (Irvine, CA, USA). Phloroglucinol (≥98%) was obtained from Beijing Solarbio Science and Technology Co., Ltd. (China). Mesalazine (MES) was purchased from the Sunflower Pharmaceutical Group Jiamusi Luling Pharmaceutical Co., Ltd. (Jiamusi, China). The enzyme-linked immunosorbent assay (ELISA) detection kits for IL-10, IL-1β, interferon-γ (IFN-γ) and TNF-α were purchased from Dakewe Biotech Co., Ltd. (Shenzhen, China). Enhanced bicinchoninic acid (BCA) protein assay and colorimetric terminal-deoxynucleoitidyl transferase-mediated nick end labeling (TUNEL) apoptosis assay kits were purchased from Beyotime Biotechnology Co., Ltd. (Shanghai, China). Primary antibodies and respective secondary antibodies were purchased from Servicebio Technology Co., Ltd. (Wuhan, China). Enhanced chemiluminescence (ECL) kit was purchased from Bio-Rad Laboratories, Inc. (Hercules, Cal, USA).

SJE was prepared as described previously Che et al. under review. Briefly, *Saccharina japonica* was ground into powder using a mixer grinder. The powdered Saccharina japonica (500 g) was extracted in 5 L of 85% ethanol (v/v) twice at 40°C temperature. After suction filtration, the filtrate was concentrated using a vacuum rotary evaporator. The residues were store at 4°C overnight and then filtered with suction pump to remove the precipitated mannitol. Finally, a freeze-dryer was used to obtain the SJE.

### Animals and Experimental Design

**Figure 1:**
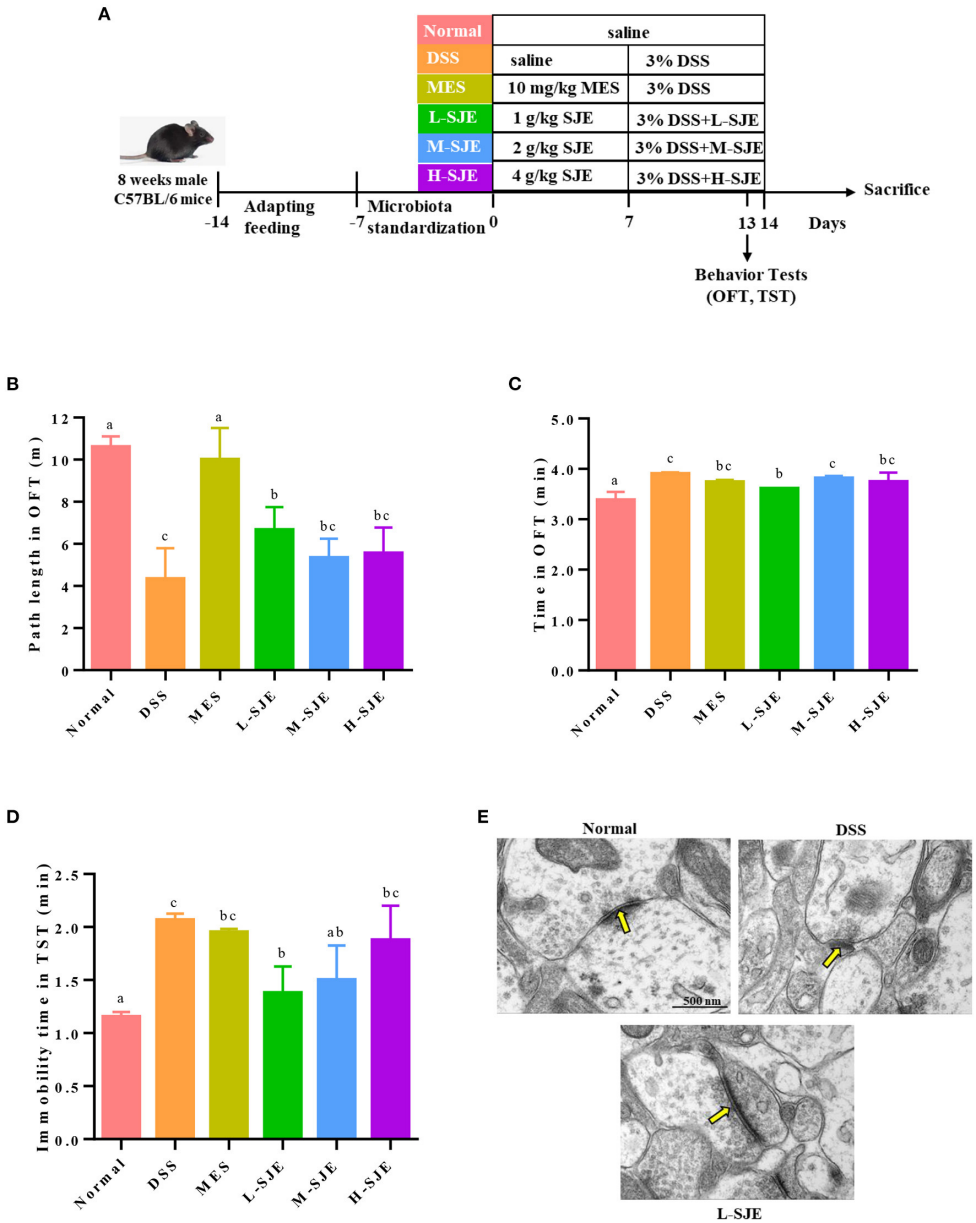
Effects of SJE on depression-/anxiety-like behavior and synapse ultrastructure in the brain of DSS-induced mice. **(A)** Experimental schedule of animal treatments and behavioral tests. **(B)** Path length and **(C)** time spent in the outer zones by mice in open field test (OFT). **(D)** Immobility time of mice in tail suspension test (TST). **(E)** Transmission electron microscopy (TEM) representative photomicrographs of mice brain. Data are presented as mean ± SEM, *n* = 8. All statistical tests were conducted using one-way analysis of variance (*post-hoc* test: Duncan) and values designated by different letters were statistically different (*p* < 0.05).

Male C57BL/6 mice (8 weeks old) with the body weight (BW) of 20–22 g, were purchased from Qingdao Daren Fortune Animal Technology Co., Ltd (Qingdao, China). All the mice were housed at 22–25°C temperature, 55 ± 5% relative humidity, and 12/12 h light/dark cycle. After 1 week of adaptive feeding and microbiota standardization, all the mice were randomly divided into 6 groups (*n* = 8): (i) Normal (normal) group; (ii) DSS (DSS treatment) group; (iii) MES (MES treatment [10 mg/kg of BW] + DSS) group; (iv) L-SJE (SJE treatment [1 g/kg BW] + DSS) group; (v) M-SJE (SJE treatment [2 g/kg BW] + DSS) group; and (vi) H-SJE (SJE treatment [4 g/kg BW] + DSS). The feeding pattern was consistent with our previous study (20). Briefly, in the third week, the mice in Normal and DSS groups only received saline, those in MES group received MES dissolved in saline, and the other groups received different doses of SJE. Then all the mice, except those in the Normal group, were allowed freely to drink water with DSS (3%), and the treatment groups received different doses of SJE once daily in the fourth week. All the animal treatments were approved by the Animal Ethics Committee of Qingdao University of Science and Technology (Approval No. SYXK2020-0422). The details of experimental design are shown in Figure 1A.

After fasted for 12 h, all animals were sacrificed by cervical dislocations under anesthesia. The brain tissues were stored in liquid nitrogen for short-term storage, and transferred to −80°C refrigerator.

### Behavioral Tests

All the behavioral tests were performed on day 13 and 14 in a sound-proof room with a neutral environment.

### Open Field Test (OFT)

As previously described, all the mice were tested separately in a device, consisting of a black square substrate (40 × 40 cm) and black walls (40 cm) placed in the corners (21, 22). After 2 min of acclimation, the free movements of mice were tracked using a video computer system for up to 4 min. The indicators for depressive behavior of mice included their traveling path and time spent in outer zones.

### Tail Suspension Test (TST)

The mouse tail was fixed with a clip about 1 cm from the end and hung upside down about 20 cm above the ground. After a period of activity, the mice developed intermittent quiescence and showed disappointment. The immobility time of the mice was recorded during the last 4–6-min of test (23).

### Transmission Electron Microscopy (TEM) Measurements

The ultra-structures of synapses were assessed using TEM. The brain tissues were collected and fixed in 2.5% phosphate-buffered glutaraldehyde for 24 h at room temperature. Then, the tissue samples were prepared for TEM examination routinely (24). Briefly, the samples were post-fixed in 1% osmium tetroxide and then dehydrated with ethanol and acetone in a graded series. After resin embedding, the embedding models were taken out after incubation at 65°C for 48 h. Then the blocks were cut into 70-nm thin sections using ultra-microtome (Leica, Weztlar, Germany), and the sections were passed through 150 mesh cuprum grids. After staining with 2% uranium acetate, 2.6% lead citrate, and CO_2_ for 8 min, dry overnight at room temperature. The sections were viewed at a magnification of 25,000 × under a TEM (H-7650, Hitachi, Japan) with an accelerating voltage of 80 kV.

### ELISA

The brain tissue samples were homogenized in saline solution (1:9 w/v) on ice. The homogenate was centrifuged (10,000 × g, 4°C) for 15 min to collect the supernatant. The enhanced BCA protein assay kit was used to detect the concentration of total proteins. The protein concentrations of TNF-α, IL-1β, IFN-γ, and IL-10 in the supernatants were detected using their corresponding commercial Invitrogen ELISA kits. Microplate Reader Spark 20 M (Tecan, Männedorf, CH) was used to measure the optical density (OD). The concentrations of inflammatory cytokines were presented in pg per mg protein.

### Western Blotting

The brain tissues were homogenized and the total protein contents were extracted following the manufacturer's instructions. The protein lysates were separated using 12% (sodium dodecyl sulfate-polyacrylamide gel electrophoresis) SDS-PAGE and transferred onto polyvinylidene fluoride (PVDF) membranes (Bio-Rad Laboratories, Hercules, Cal, USA). Afterward, the membranes were blocked with 5% skim milk and incubated overnight with the primary antibodies of TLR4, NF-κB-p65, NLRP3, and β-actin at 4°C. Finally, after incubation with the respective secondary antibodies for 2 h, the signals were detected using an ECL kit and analyzed using a ChemiDoc Touch Chemiluminescence imaging system (Bio-Rad Laboratories, Hercules, Cal, USA).

### Immunohistochemistry (IHC) Staining

The sections paraffin-embedded sections were dewaxed, rinsed with distilled water, and repaired with citric acid (pH 6.0) antigen repair solution. After repairing, the sections were rinsed with phosphate buffer saline (PBS) (pH 7.4) three times for 5 min and blocked with blocking buffer (3% bull serum albumin in PBS) at room temperature for 30 min. The brain tissue sections were then incubated with the primary antibodies of Caspase-1, apoptosis-associated speck-like protein (ASC) and ionized calcium-binding adaptor molecule-1 (Iba-1) in a humidified chamber at 4°C overnight. The tissue sections were then incubated with corresponding secondary antibodies at room temperature for 50 min. After rinsing with PBS, the sections were stained with chromogenic reagent diaminobezidin (DAB) and hematoxylin. Finally, light microscope (E100, Nikon, Japan) and imaging system (Nikon DS-U3, Nikon, Japan) were used to observe the tissue sections and capture images. The immunohistochemistry score (IHC score) were evaluated by image analysis system (Image-Pro Plus 6.0, Media Cybemetics, USA) according to the positive area and intensity. IHC score = ∑(pi×i) = (percentage of weak intensity area ×1)+(percentage of moderate intensity area ×2)+(percentage of strong intensity area ×3). pi: percentage of pixel area of positive signal; i: positive level. The larger the H-score value, the stronger the comprehensive positive intensity.

### Terminal-Deoxynucleoitidyl Transferase Mediated Nick End Labeling (TUNEL) Assay

For the TUNEL assay, positive cells in brain sections were detected using the commercial colorimetric TUNEL apoptosis assay kit, according to the manufacturer's instructions.

### Determination of Oxidative Response

The freshly excised tissues were rinsed with saline, homogenized in tissue lysis buffer, and then centrifuged at 10,000 g and 4°C for 15 min. The changes in the enzymatic activities of brain superoxide dismutase (SOD) and malonicdialdehyde (MDA) were measured using their corresponding kits (Jiancheng Bio., Nanjing, China) and presented as pictograms of U/mg and nmol/mg protein, respectively.

### Statistical Analyses

Statistical analyses were performed using GraphPad Prism 8. All the data were presented as means ± standard deviation. Differences between the mean values were evaluated using one-way analysis of variance (*post-hoc* test: Duncan). The *p* < 0.05 and <0.01 were considered statistically significant and highly statistically significant, respectively.

## Results

### Effects of SJE on Depression-/Anxiety-Like Behavior and Synapse Ultrastructure in the Brain of DSS-Induced UC Mice

In OFT, as compared to normal mice, the DSS-induced mice showed significant decrease in their path length and increase the time spent in outer zones (Figures 1B,C). On contrary, the MES and L-SJE group mice remarkably alleviated these behavioral abnormalities. In TST, the DSS-induced mice had longer immobility time as compared to the normal mice (Figure 1D), while those in the MES, L-SJE, and M-SJE showed remarkably decrease in the immobility time.

The synapse ultrastructure, observed by TEM, showed that the DSS-induced mice exhibited thinner and shorter postsynaptic densities (PSDs) as compared to normal group (Figure 1E). Considering the prominent effects of L-SJE in ameliorating the depression/anxiety-like behavior, the synapse ultrastructure of the L-SJE group mice brain tissues was observed. The PSDs in L-SJE group mice recovered to a relatively normal level. These results demonstrated that the SJE could alleviate depression/anxiety-like behavior and maintain synaptic plasticity in the brain of DSS-induced mice.

### Effects of SJE on the Activation of Microglia and Inflammatory Cytokine

**Figure 2:**
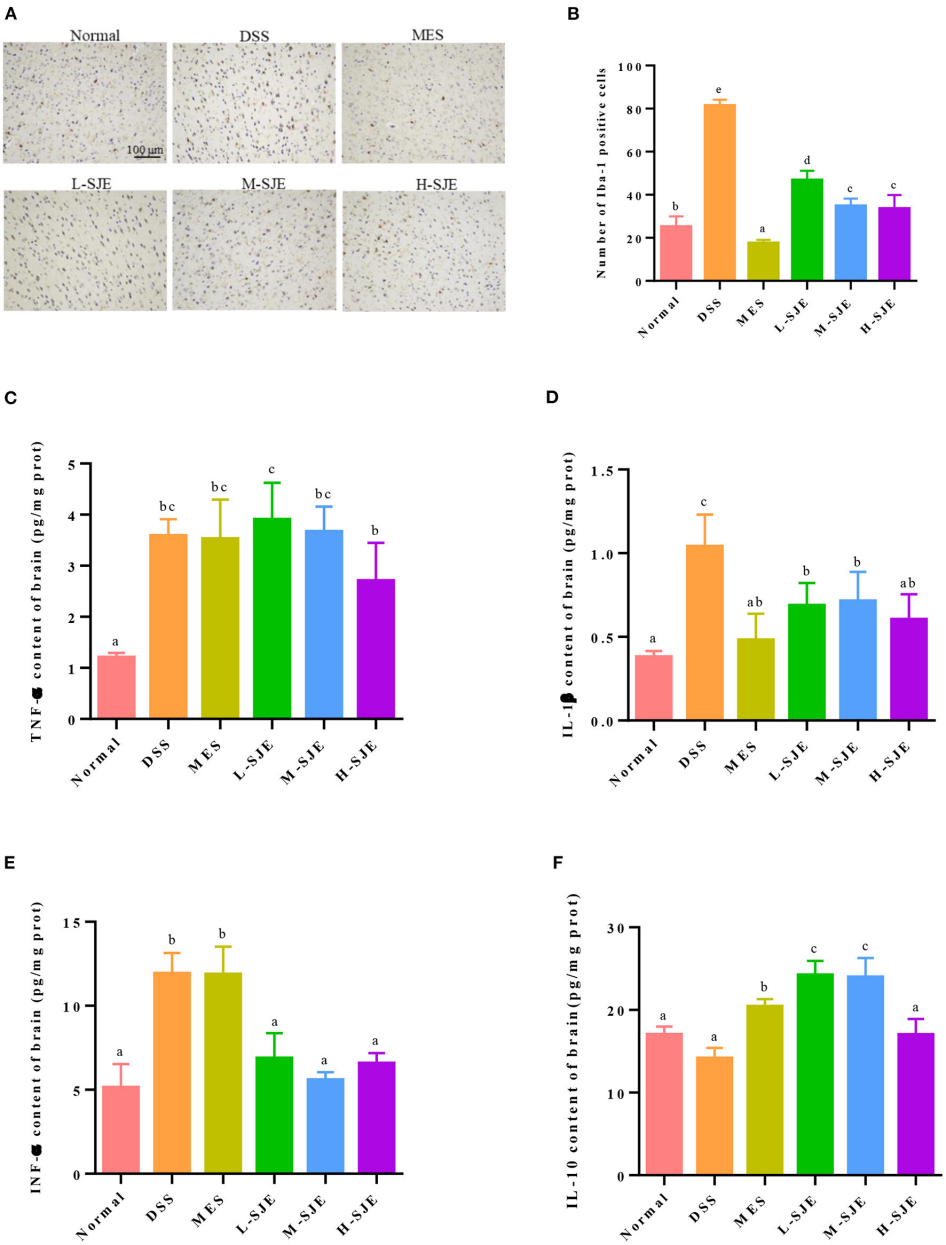
Effects of SJE on the activation of microglia and inflammatory cytokine. **(A)** Representative immunohistochemical images of microglial activation marker, ionized calcium-binding adaptor molecule-1 (Iba-1), in mice brain (200X). **(B)** Statistical numbers of Iba-1 positive cells in mice brain. Expression of **(C)** TNF-α, **(D)** IL-1β, **(E)** IFN-γ, and **(F)** IL-10 in mice brain measured by ELISA. Data are presented as mean ± SEM, *n* = 8. All statistical tests were conducted using one-way analysis of variance (*post-hoc* test: Duncan) and values designated by different letters were statistically different (*p* < 0.05).

As shown in Figures 2A,B, the IHC results indicated that the Iba-1 expression was higher in DSS-induced mice as compared to the normal mice, which indicated the presence of more activated microglia after DSS exposure. On contrary, the numbers of activated microglia reduced after MES and SJE treatments. These results were paralleled by alterations in inflammatory cytokines in DSS-induced mice (Figures 2C–F). For the proinflammatory cytokines TNF-α, IL-1β, and IFN-γ, the DSS-induced mice showed a significant increase in their expression by 1.98-, 1.73-, and 1.32-folds, respectively. For the anti-inflammatory cytokines, the DSS-induced mice showed a 16.9% reduction in the IL-10 expression. These results indicated that the DSS exposure could significantly increase the levels of proinflammatory cytokine and decreased those of anti-inflammatory cytokines in the brain, showing an immunomodulatory dysfunction. Furthermore, the MES and SJE treatment could abolish the DSS-induced aberrant changes in inflammatory cytokines except for TNF-α to certain extents.

### Effects of SJE on the Activation of TLR4 Signaling Pathway

**Figure 3:**
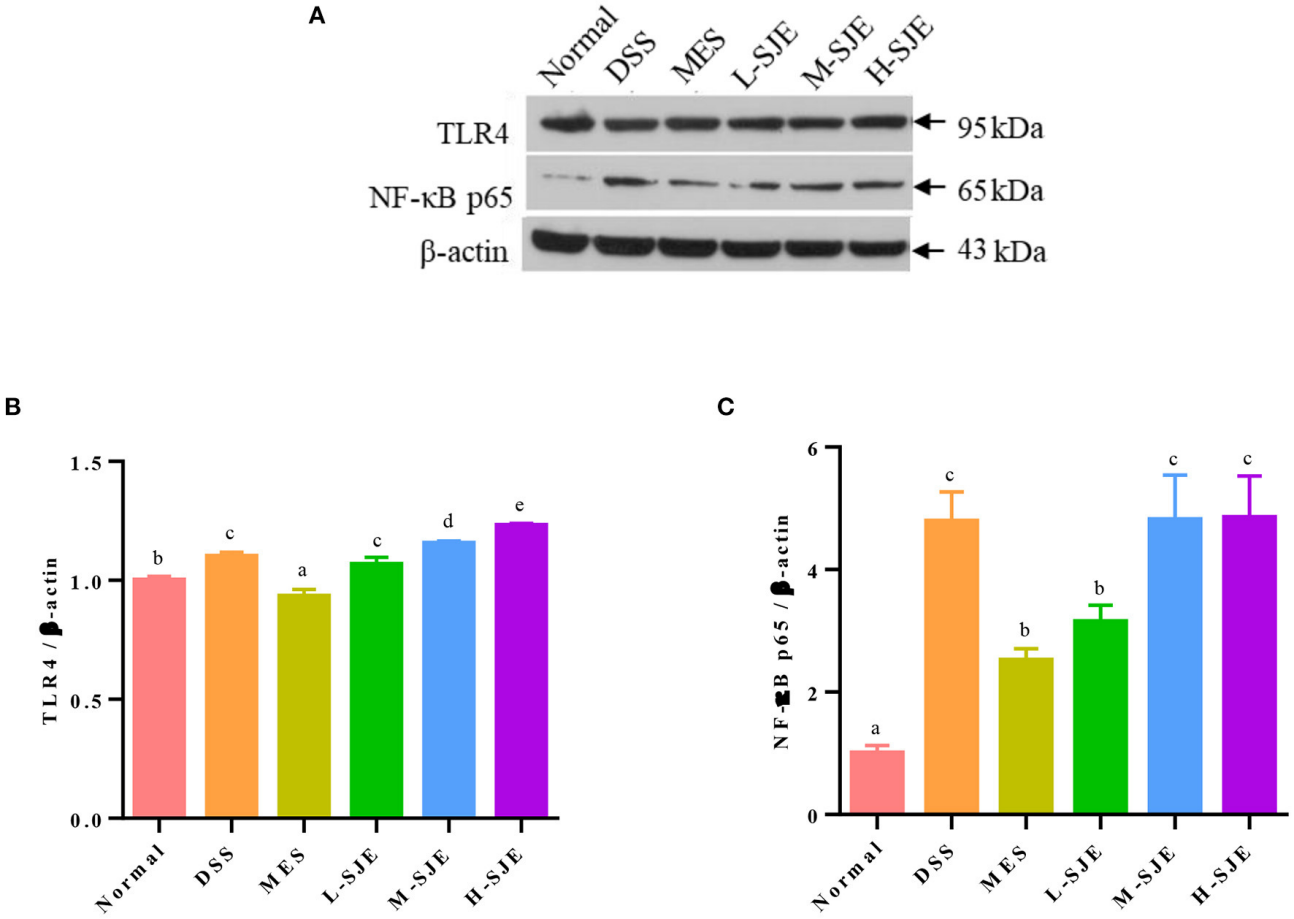
Effects of SJE on the TLR4 signaling pathway activation. **(A)** Representative western blotting images of TLR4, NF-κB p65, and β-actin. **(B,C)** Qualitative analysis of TLR4 and NF-κB-p65. Data are presented as mean ± SEM, *n* = 5. All statistical tests were conducted using one-way analysis of variance (*post-hoc* test: Duncan) and values designated by different letters were statistically different (*p* < 0.05).

The protein expression levels of TLR4 and NF-κB-p65 in mice brains increased significantly by 0.083- and 3.7-fold after DSS exposure and recovered significantly after MES treatment (Figure 3). As compared to DSS group, the L-SJE had a significant down-regulatory effect on NF-κB pathway but not on the TLR4 pathway, while the M-SJE and H-SJE had no significant effects on both the proteins.

### Effects of SJE on the Activation of NLRP3 Inflammasome Pathway

**Figure 4:**
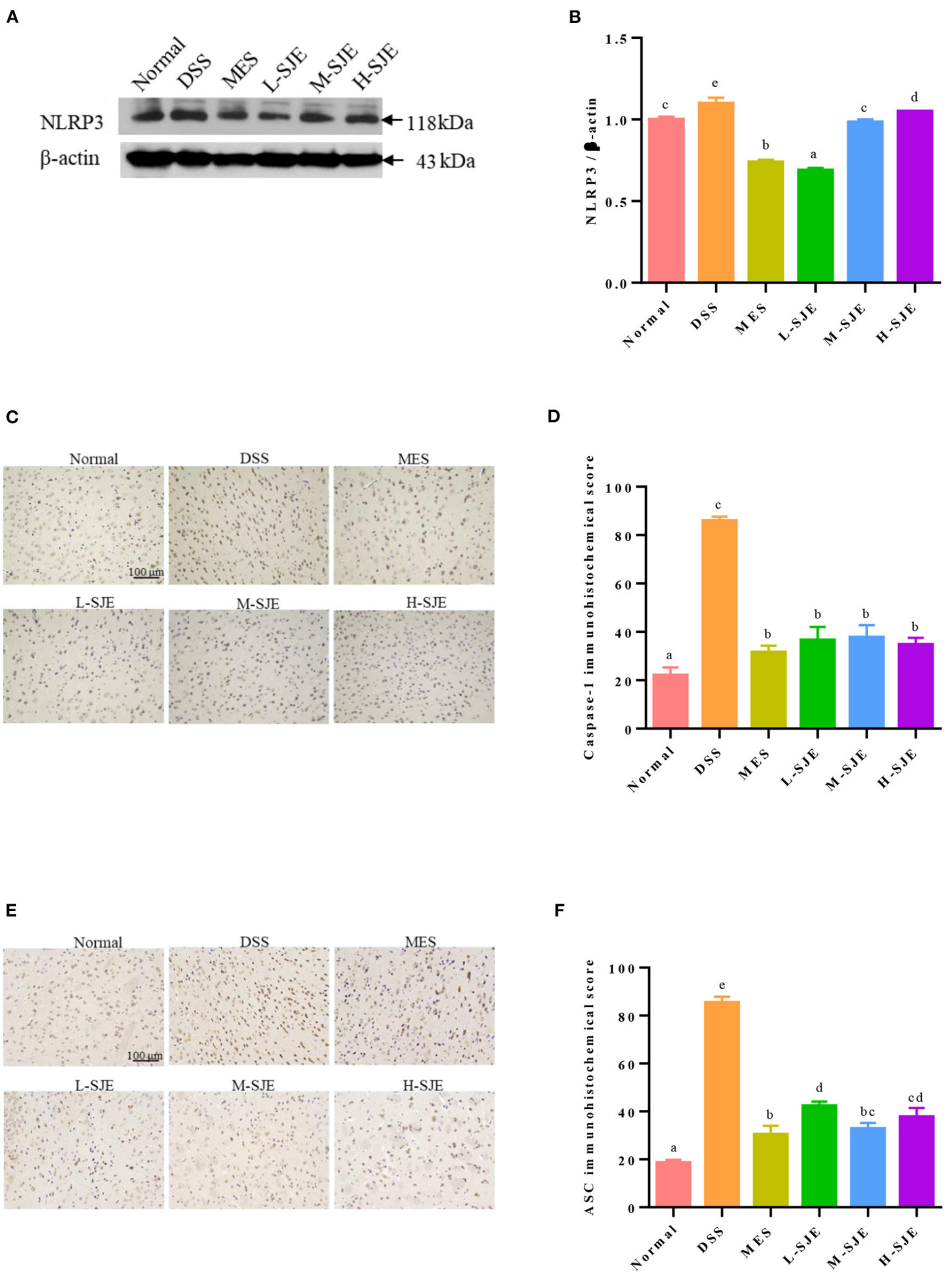
Effects of SJE on the NLRP3 inflammasome pathway activation. **(A)** Representative western blotting images of NLRP3 and β-actin. **(B)** Qualitative analysis of NLRP3. Immunohistochemical analysis and scores of **(C,D)** Caspase-1 and **(E,F)** ASC. Data are presented as mean ± SEM, *n* = 5. All statistical tests were conducted using one-way analysis of variance (*post-hoc* test: Duncan) and values designated by different letters were statistically different (*p* < 0.05).

As shown in Figures 4A,B, the NLPR3 proteins levels in the mice brain significantly increased after DSS induction. The MES and SJE treatment could significantly modulate the NLRP3 proteins levels as compared to their over-expressed levels in DSS group, where the L-SJE group showed the lowest NLRP3 protein expression. These results were consistent with the expression levels of ASC and Caspase-1 (Figures 4C–F). As compared to the normal mice, a significant increase in the IHC scores of ASC and Caspase-1 was observed in DSS mice. The MES and SJE treatments remarkably inhibited their protein expression.

### Effects of SJE on Cerebral Oxidative Stress

**Figure 5:**
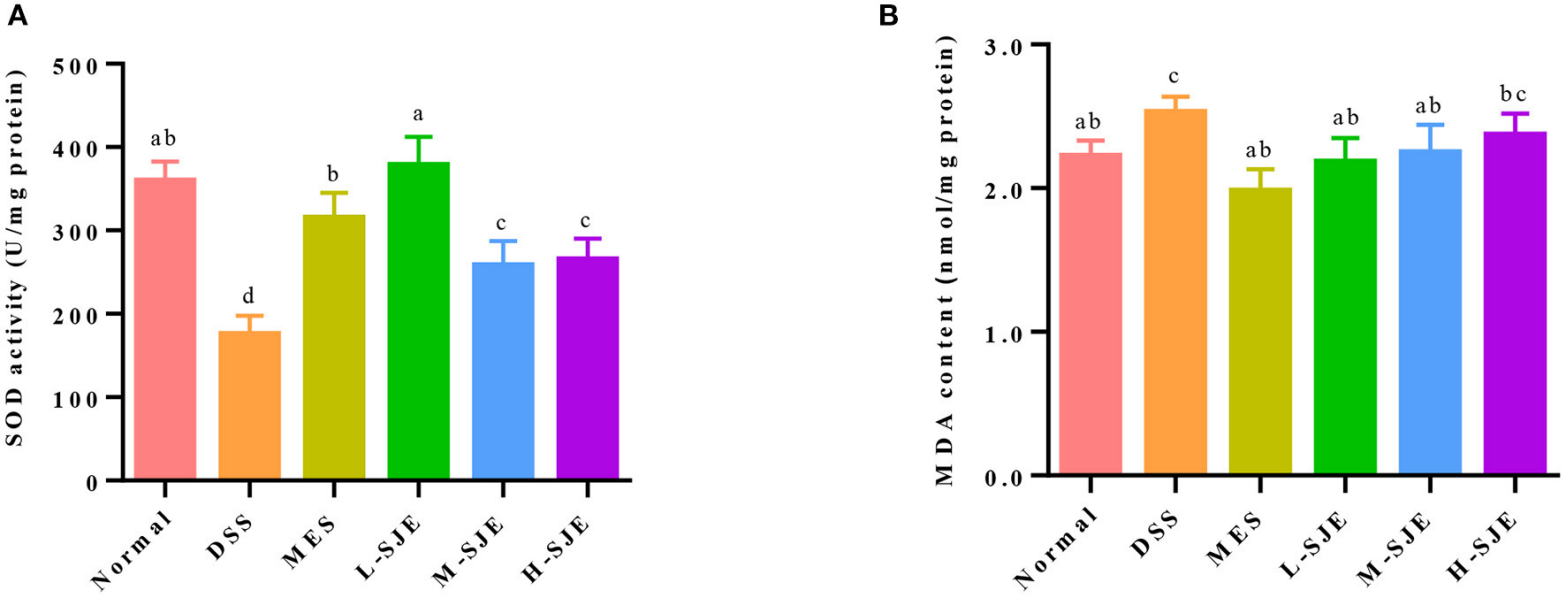
Effects of SJE on cerebral oxidative stress. **(A)** SOD activity. **(B)** MDA content. Data are presented as mean ± SEM, *n* = 5. All statistical tests were conducted using one-way analysis of variance (*post-hoc* test: Duncan) and values designated by different letters were statistically different (*p* < 0.05).

In this study, SOD activity in the DSS group was significantly reduced by 51.2% as compared to the normal group but significantly increased in the MES group and SJE groups, especially in the L-SJE group, where it was almost restored to the normal level (Figure 5A). A significant increase in the MDA level was observed in the brain of DSS-induced UC mice as compared to the normal group but the MES and L-SJE reversed that change (Figure 5B).

### Effects of SJE on Cerebral Apoptosis

**Figure 6:**
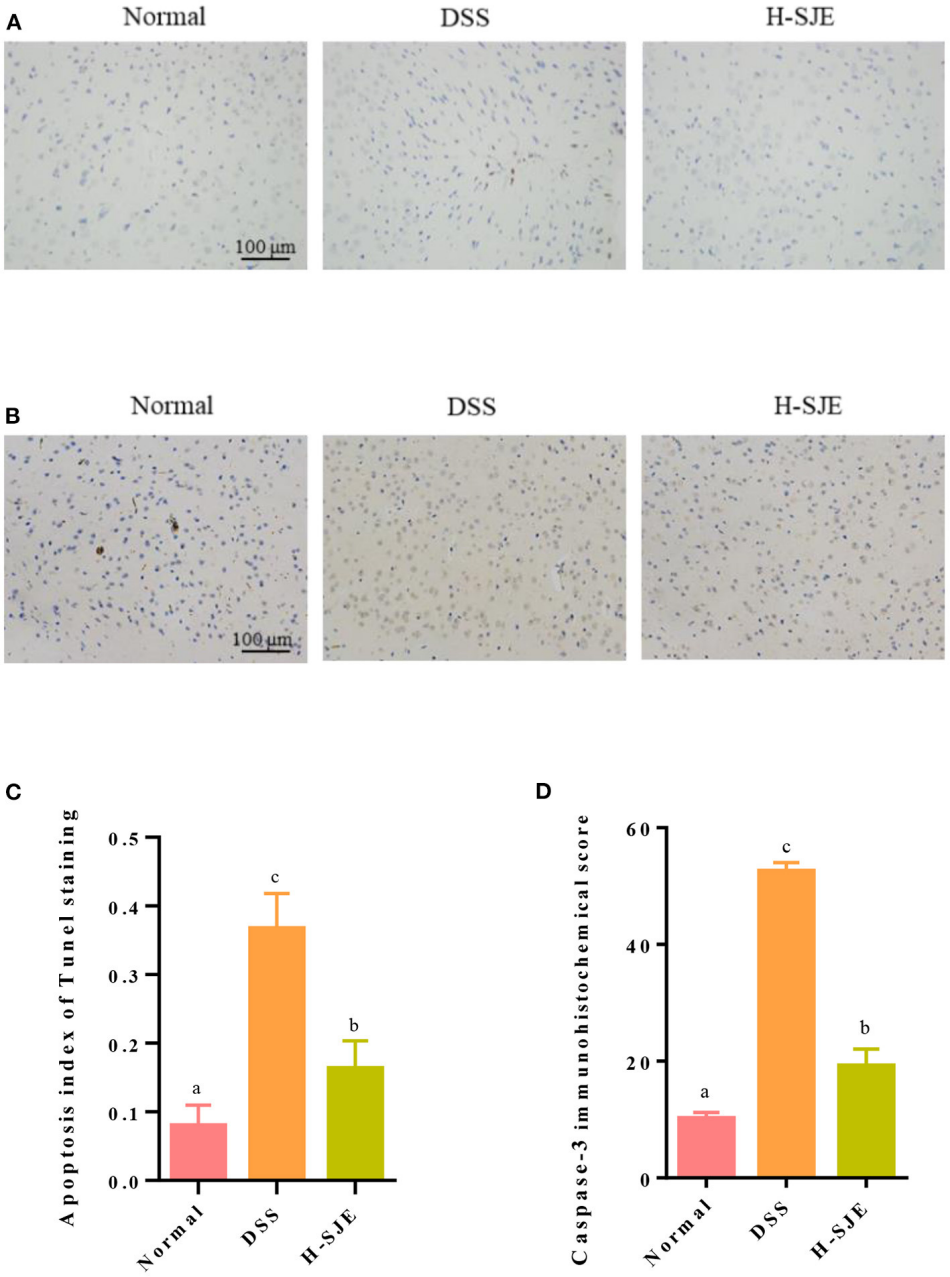
Effects of SJE on cerebral apoptosis. **(A)** Representative photomicrographs, demonstrating the detection of apoptotic cells by TUNEL stain (200X). **(B)** Representative immunohistochemical photomicrograph of Caspase-3 (200X). **(C)** Analysis of apoptosis index and **(D)** immunohistochemical score of Caspase-3 score. Data are presented as mean ± SEM, *n* = 5. All statistical tests were conducted using one-way analysis of variance (*post-hoc* test: Duncan) and values designated by different letters were statistically different (*p* < 0.05).

In this study, as compared to the normal group, significantly a greater number of TUNEL positive (stained brown) cells were observed in the brain of DSS-induced mice and the high dose of SJE significantly reduced this effect (Figures 6A,C). Caspase-3, an executive factor of apoptosis, presented similar results in the present study (Figures 6B,D). These results indicated that the DSS significantly induced apoptosis in the mice brain but the H-SJE could significantly recover apoptosis to normal levels.

## Discussion

UC is often accompanied by psychiatric disorders, such as depression and anxiety (25). Meanwhile, the patients with psychiatric disorders show similar changes in their gut microbiota, such as reduced abundance of Clostridiales, Firmicutes, and Clostridia, and increased abundance of Bacilli, Proteobacteria, and Gammaproteobacteria (26). Our previous paper reported that the similar imbalance of gut microbiota in DSS mice, and SJE could resume the integrity of intestinal barrier and reshape the gut microbiota (20). However, whether SJE also ameliorates the depression and anxiety induced by UC and the potential mechanism is unclear. Based on the disturbance of microbiota-gut-brain axis, it is proposes that SJE may regulate brain neurochemistry and behavior by regulating the gut microbiota, their metabolites and immune mediators through central nervous system (CNS) communication in the present study. The results indicated that the SJE could inhibit inflammatory response in the brain of DSS-induced mice by regulating the TLR4/NLRP3 signaling pathway, while suppressing apoptosis and oxidative stress, thereby improving the depressive/anxiety-like behavior.

DSS treatment damages colonic epithelial cells and causes intestinal inflammation, including elevated levels of inflammatory cytokines, by epithelial cytotoxicity, increased intestinal permeability, and macrophage activation. Inflammatory factors are transported through the circulatory system, formulating inflammation in brain and thereby inducing depression or anxiety (27, 28). OFT and TST are three widely-used tests to evaluate the depressive/anxious behavior. OFT evaluates locomotion, exploratory activity, and anxiety-like behaviors in new environmental conditions, while TST evaluate despair in the specific states of depressive immobility (29). In this study, the SJE treated group, especially the low-dose group, significantly alleviated the DSS-induced depressive/anxious behavior. Some plant extracts, such as *Schisandra Chinensis* (30), and *okra seeds* (4) have also shown similar effects and suggested that the major bioactive substances were polyphenols, flavonoids, lignans, saponins and other small molecules. SJE also contains a variety of bioactive substances, such as brown algae polyphenols and lignans, which have potential therapeutical effects on the DSS-induced depressive behavior in mice. The behaviors of depression/anxiety disorders are often accompanied by ultrastructure changes of brain tissues, such as changes in synaptic plasticity. Yan et al. reported that PSD expression was abnormal in the brain tissue of depressed patients, as evidenced by reduced thickness and length and blurred synaptic structures (31). The low dose of SJE could improve synaptic plasticity (Figure 1E). Similarly, lycopene presented similar effects on the DSS-induced UC mice (23).

Psychiatric disorders are closely associated with the activation of immune/inflammatory response, which is manifested by the activation of macrophages (such as microglia) in CNS, inducing the release of multiple inflammatory cytokines and creating a “waterfall” effect (32). The above cascade of responses has been widely reported in DSS-induced UC mice (33, 34). In this study, the activation of microglial cells and over-expression of multiple inflammatory cytokines were found in the DSS group, while the SJE treatment alleviated these changes (Figure 2). Combined with the literature, it was inferred that SJE may mediate inflammatory factors to influence depressive/ anxious-like behaviors in two ways. Pro-inflammatory cytokines directly can increase the neuro-modulatory cytokine signaling by “leaking” through the subventricular organs across the blood-brain barrier (BBB); or the cytokines can stimulate afferent pathways, causing behavioral changes (35). For example, by affecting neurotransmitters, such as 5-hydroxytryptamine, dopamine, and norepinephrine (NE), the cytokines can influence neural circuits, thereby modulating behavior (36, 37).

In CNS, TLR4 is one of the most widely studied proteins, which is mainly expressed in microglia and its activity can affect the depression-like behavior (38). TLR4 promotes the nuclear translocation of NF-κB mainly through the activation of factor-dependent myeloid differentiation 88 (MyD88) and non-dependent MyD88 pathways. The activation and translocation of NF-κB to nucleus stimulates the release of other pro-inflammatory cytokines, including TNF-α. and initiates inflammasome assembly. NLRP3 recruits a downstream junction protein, ASC, and an effector protein, Caspase-1, to assemble and form NLRP3 inflammasome (39). TLR4/NLRP3 signal activation can polarize microglia to Ml phenotype and induce a pro-inflammatory response (40). In this study, the up-regulation of TLR4, NF-κB p65, NLRP3, ASC, and Caspase-1 were observed in the brain tissues of DSS-induced mice (Figures 3, 4). However, these key proteins expression levels in SJE treatment groups, especially the L-SJE group, were down-regulated as compared to the DSS group except that of TLR4, which indicated that more key proteins, such as MyD88, IL-1 receptor-associated kinase (IRAK), and TNF receptor-associated factor 6 (TRAF 6), should be analyzed to evaluate the activation of the TLR4 signaling pathway. Furthermore, SJE might also inhibit the activation of the NLRP3 inflammasome by other pathways, such as autophagy (41). Multiple plant extracts, such as green tee extract (42, 43), gape seed extract (44), loquat fruit extract (45), and Radix Polygalae extract (41), have shown anti-inflammatory activity through TLR4/NLRP3 signaling pathway. Moreover, it has been reported that the NLRP3 inflammasome can further induce secondary signaling, lysosomal instability, and reactive oxygen species (ROS) production, thereby mediating apoptosis (46).

The oxidative stress and apoptosis have been widely demonstrated to be associated with depression or anxiety disorders in addition to inflammatory/immune responses (6). Brain is particularly vulnerable to oxidative damage due to a high consumption of oxygen and long-chain polyunsaturated fatty acid contents (6). The oxidative damage can be indicated by the activity of antioxidant enzymes, such as SOD and catalase, and the content levels of MDA, a product of lipid peroxidation. In this study, the decreased activity of SOD and increased MDA contents were found in DSS-induced UC mice, which could be alleviated by SJE treatment (Figure 5). A previous study has also reported that the ethanol extracts of brown algae *Undaria pinnatifida* (47), *Eisenia arborea*, and *Macrocystis pyrifera* (48) exhibit favorable antioxidant and anti-inflammatory properties. Moreover, the cascade events of oxidative stress trigger the apoptosis through mitochondria (49). In the brain of DSS-induced mice, apoptosis often occurs concomitantly, as evidenced by the increase in TUNEL-positive cells, over-expression of Caspase-3, down-regulation of anti-apoptotic effects, and up-regulation of pro-apoptotic effects (33, 50). In this study, similar results were observed in the DSS-induced mice, and the high dose of SJE treatment could alleviate this effect (Figure 6). The data suggested the antioxidant and anti-apoptotic properties of SJE exhibited a potential relationship with its antidepressant effects.

## Conclusion

In conclusion, the above results indicated that the SJE could relieve the depression/anxiety-like behaviors in DSS-induced mice and maintained the synaptic plasticity and resting state of microglia. Moreover, SJE could suppress inflammatory levels by TLR4-NLRP3 signaling pathways and alleviating oxidative stress and apoptosis. Therefore, the inhibition of neuroinflammation, oxidative stress, and apoptosis might be the antidepressant mechanism of SJE, making it a potential candidate for the prevention of depressive/anxiety disorder.

## Data Availability Statement

The raw data supporting the conclusions of this article will be made available by the authors, without undue reservation.

## Ethics Statement

The animal study was reviewed and approved by Ethics Committee of Qingdao University of Science and Technology.

## Author Contributions

WX provided the project administration and funding acquisition. XD designed the research and wrote the manuscript. KL and PL executed the experiments and analyzed the data. HC, HL, LS, and XY reviewed and edited this manuscript. All authors have read and agreed to the published version of the manuscript.

## Funding

This work is supported by National Key R&D Program of China (2018YFD0901105).

## Conflict of Interest

The authors declare that the research was conducted in the absence of any commercial or financial relationships that could be construed as a potential conflict of interest.

## Publisher's Note

All claims expressed in this article are solely those of the authors and do not necessarily represent those of their affiliated organizations, or those of the publisher, the editors and the reviewers. Any product that may be evaluated in this article, or claim that may be made by its manufacturer, is not guaranteed or endorsed by the publisher.
